# Transcriptome analysis of human tissues and cell lines reveals one dominant transcript per gene

**DOI:** 10.1186/gb-2013-14-7-r70

**Published:** 2013-07-01

**Authors:** Mar Gonzàlez-Porta, Adam Frankish, Johan Rung, Jennifer Harrow, Alvis Brazma

**Affiliations:** 1 European Molecular Biology Laboratory - European Bioinformatics Institute, EMBL-EBI, Wellcome Trust Genome Campus, Hinxton, Cambridge, CB10 1SD, United Kingdom; 2 Wellcome Trust Sanger Institute, Wellcome Trust Genome Campus, Hinxton, Cambridge, CB10 1SA, United Kingdom

**Keywords:** splicing, transcriptome, gene expression, RNA-seq

## Abstract

**Background:**

RNA sequencing has opened new avenues for the study of transcriptome composition. Significant evidence has accumulated showing that the human transcriptome contains in excess of a hundred thousand different transcripts. However, it is still not clear to what extent this diversity prevails when considering the relative abundances of different transcripts from the same gene.

**Results:**

Here we show that, in a given condition, most protein coding genes have one major transcript expressed at significantly higher level than others, that in human tissues the major transcripts contribute almost 85 percent to the total mRNA from protein coding loci, and that often the same major transcript is expressed in many tissues. We detect a high degree of overlap between the set of major transcripts and a recently published set of alternatively spliced transcripts that are predicted to be translated utilizing proteomic data. Thus, we hypothesize that although some minor transcripts may play a functional role, the major ones are likely to be the main contributors to the proteome. However, we still detect a non-negligible fraction of protein coding genes for which the major transcript does not code a protein.

**Conclusions:**

Overall, our findings suggest that the transcriptome from protein coding loci is dominated by one transcript per gene and that not all the transcripts that contribute to transcriptome diversity are equally likely to contribute to protein diversity. This observation can help to prioritize candidate targets in proteomics research and to predict the functional impact of the detected changes in variation studies.

## Background

Although there are fewer than 22,000 protein coding genes known in the human genome, they are transcribed into over 140,000 different transcripts (Ensembl release 66 [1]), over 65% of which have protein coding potential and thus may contribute to protein diversity. Recently, applications of high throughput sequencing to RNA, known as RNA-seq [2], have opened new avenues for the study of transcriptome composition [3]. RNA-seq is based on sequencing short fragments, thus making the precise reconstruction of full-length transcripts a difficult task; nevertheless, several methods have been developed to deconvolute transcript abundance [4-6]. Significant evidence has accumulated showing that approximately 95% of multiexon genes have more than one alternative splice-form expressed (for example, [4,7-9]) and that transcript expression is regulated [10,11]. On the other hand, focusing on EST data, Taneri *et al. *[12] predicted that there is a single dominant transcript per gene in primary tissues. Recently, the ENCODE project [13] showed that indeed, in cell lines most genes have a major transcript, although at the same time noted that 'genes tend to express many transcripts simultaneously, and as the number of annotated transcripts per gene grows, so does the number of expressed transcripts'. However, despite these observations, it is still not clear if and to what extent major transcripts are dominating the transcriptome and what proportion of the transcript diversity is likely to contribute to protein diversity. In addition, given the notable differences in gene expression between primary tissues and cell lines [11,14], transcriptome analysis in cell lines can be extended to primary tissues only to some extent.

Here we intend to characterise the potentially coding transcriptome from a functional perspective. By focusing on protein coding genes, we show that in primary tissues almost 85% of the total mRNA from protein coding loci comes from major transcripts (76% in cell lines). It is important to highlight that these major transcripts are not always the longest possible for the gene (40% of the major transcripts in primary tissues and 30% in cell lines are not the longest annotated), nor always include the longest CDS (Coding DNA Sequence; approximately 50% of the cases in both tissues and cell lines). For instance, we identified the *AES *gene (amino-terminal enhancer of split), for which we detect a ubiquitous major transcript that is shorter than the current reference. We also show that the ratio of the number of expressed transcripts to genes in primary tissues is on average 1.12 (that is, just over one transcript per gene). We further distinguish between: (1) major transcripts - the transcript with the highest expression level within a given gene; and (2) dominant transcripts - a major transcript that is expressed at a considerably higher level than any minor transcripts of the gene. We show that most protein coding genes in most conditions have one dominant transcript, for example, for almost 80% of the expressed genes in primary tissues the major transcript is at least twice as abundant as the next one.

We further observe that about half of the ubiquitously expressed genes (*n *= 4,801) have the same major transcript across all the 16 tissues studied here. We do, however, detect switch events for approximately 35% of the genes, where the dominant and minor transcripts switch between different tissues, while the total expression level of the gene changes comparatively little. In around 100 genes we observe such a strong change that we can hypothesise that the different transcripts are likely to be translated into different proteins. One example is the *MBP *gene (Myelin Basic Protein), which is a major protein constituent of the myelin sheath. A shorter brain specific form has been detected by this analysis and has been highlighted recently in the literature [15]. Finally, we observe that for almost 20% of the studied protein coding genes (*n *= 18,450) the major transcript does not code for a protein, and this percentage is considerably higher in nucleus than in cytosol. Half of the noncoding major transcripts can be explained by a retained intron, typically located towards the 3'-end of the transcript.

We perform the analyses using three different computational methods [4-6], and additionally, where sufficient coverage exists, we assess the alternative transcript abundances directly from the reads spanning unique exon junctions. We also use simulated data [16] to confirm that the methods can reliably distinguish between two hypothetical alternative scenarios - one dominant transcript per gene *vs*. several transcripts per gene expressed to similar levels. All those methods produce a consistent outcome, indicating robustness of the conclusions presented here.

Overall, our results show that, despite of the diversity of the transcriptome, most protein coding genes have one dominant transcript, which, when combined, comprise most of the potentially coding mRNA transcriptome. Correlation between transcriptome and proteome is not straightforward, with the best estimates pointing at a range of 58% to 63% correlation [17]. However, the strong overlap with a set of isoforms that are independently predicted to be translated into proteins, together with the clear separation in expression levels between major and minor transcripts, add support to the hypothesis that the dominant transcripts are likely to be the main contributors to the proteome. Thus, our findings may help proteome analysis by prioritising the candidate proteins that are more likely to be present in a given sample. At the same time, identification of changes in the major transcript across conditions can lead to relevant clinical findings (for example, [18]) and may also be used to predict the functional impact of the detected changes in variation studies. Nevertheless, this does not imply that all minor transcripts do not have a biological function, since some may still be translated into proteins [19] or have a regulatory function as mRNAs [20].

## Results and discussion

Here we quantify and analyse the overall contribution of major transcripts to the potentially coding mRNA transcriptome, in comparison to minor transcripts. The datasets analysed comprise 16 primary human tissues - Illumina Body Map dataset (BM), further referred to as tissue data - and 5 ENCODE cell lines, including different cellular compartments (whole cell, cytosol and nucleus) - further referred to as cell line data (see Methods).

### Most protein coding genes express one dominant transcript

**Figure 1:**
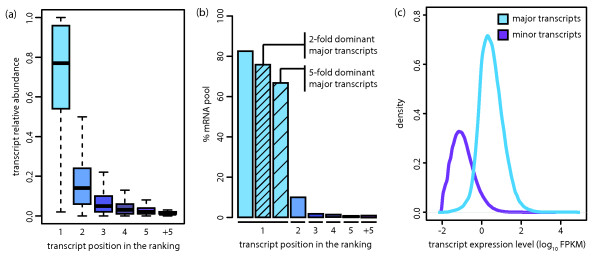
***Most protein coding genes express one predominant transcript***. (***a***) Relative abundance of the subset of transcripts in each position of the ranking for the primary tissues dataset. For each gene, transcripts were ranked based on their relative abundances. There is generally one predominant transcript over the rest. *(**b**) Percentage of the studied mRNA pool explained by each category of transcripts for the BM dataset. The mean percentage for all samples is represented here. Major transcripts represent approx*imately *85% of the studied mRNA population and were further classified into *two-*fold and *five-*fold dominant*. (**c**) Expression distribution for major and minor transcripts in the tissue dataset. We detect a total of 31,902 transcripts expressed above 1 FPKM in at least one tissue and 26,641 different major transcripts.

First, examining only the tissue data, and similarly to previous RNA-seq-based transcriptome studies, we detect more than one transcript for approximately 85% of the studied genes (83.70% to 89.95%, SD = 1.84, Additional File 2 - Table S1) and a total of 105,456 different transcripts in at least one tissue, which corresponds to approximately 90% of the studied transcripts (*n *= 117,759; see Methods). However, when quantifying all the annotated transcripts within a gene based on their relative abundance, we observe the existence of a predominant transcript for most genes in most conditions, rather than a subset of transcripts that are similarly expressed (Figure 1a, Additional File 1 - Figure S1 and Additional File 1 - Figure S2). This observation becomes even more evident when grouping transcripts by Transcription Start Site (TSS), which provides a scenario where all the different transcripts are under similar transcriptional control and where most differences in their abundance can be attributed to alternative splicing (AS; Additional File 1 - Figure S4). In the same line, we observe that the ratio of the number of expressed transcripts to genes in primary tissues is 1.12 (0.98-1.40, SD = 0.11; Additional File 2 - Table S2). Finally, we find that in the studied samples approximately 85% (79.98% to 86.49%, SD = 2.17) of the mRNA pool from protein coding loci is comprised exclusively of major transcripts (Figure 1b and Additional File 2 - Table S3). In order to address the impact of our observations at the protein level, we plotted the distribution of expression levels for both major and minor transcripts (Figure 1c) and observed that minor transcripts tend to be expressed below 1 FPKM, a threshold that has been suggested as the minimum expression required for protein detection [21-23]. In addition, we calculated the overlap of our major/minor transcript predictions with those obtained from an independent study to assess which transcripts are likely to be translated into proteins and detected a higher overlap for major transcripts (see Additional File 4 - Supplementary Results).

We quantified transcript dominance by calculating for every gene the ratio of the expression levels between the major transcript and the second most abundant one (Additional File 1 - Figure S4). Overall, we found that in the studied tissues, 79% of the genes (74.21% to 81.94%, SD = 2.16) have a two-fold dominant major transcript (that is, expressed twice as much as the second most abundant one), and that for 56% of the genes (43.39% to 61.60%, SD = 3.50) the major transcript is five-fold dominant (Table 1 and Additional File 2 - Table S4). This indicates that for most genes in a given sample there is one dominant transcript. We estimate that dominant transcripts account for most of the studied mRNA pool - 76.69% (70.04% to 80.74%, SD = 3.48) for a two-fold dominance and 67.47% (59.97% to 73.83%, SD = 4.81) for a five-fold dominance (Figure 1b). GO enrichment analysis of genes that consistently express a five-fold dominant transcript across the 16 tissues in the tissue dataset indicated that they are functionally involved in cellular respiration, protein transport, transcription and transcription regulation (Additional File 2 - Table S5). We also calculated the fraction of dominant major transcripts *vs*. non-dominant ones for different FPKM thresholds on total gene expression. The proportion of dominant major transcripts increases with higher FPKM thresholds, thus suggesting that transcriptome diversity decreases for highly expressed genes (Additional File 1 - Figure S5). Focusing on genes that tend to express several transcripts at a similar level, we identified 463 genes in the tissue dataset for which the major transcript was less than five-fold dominant in all the tissues analysed, and only 17 for a two-fold dominance threshold. GO enrichment analysis of those revealed that they are involved in RNA splicing/processing, post-transcriptional regulation of gene expression and regulation of translation (Additional File 2 - Table S6).

**Table 1:** Major transcripts tend to be predominantly expressed.

	**Expressed genes**		**Genes with a dominant major transcript**			
			**Two-fold dominance**		**Five-fold dominance**	
	
1 FPKM	10,410	56.42%	8,179	78.51%	5,864	56.22%
5 FPKM	4,671	25.32%	3,898	83.64%	3,077	66.27%
10 FPKM	2,486	13.47%	2,146	86.54%	1,794	72.60%

Average number of genes with dominant major transcripts detected in the primary tissues dataset. Different dominance ratios and gene expression thresholds were considered in the quantification.

We applied the same quantifications in cell line data, including different cellular compartments, and observed that major transcripts constitute approximately 80% of the studied mRNA pool in cytosol (77.20% to 83.66%, SD = 1.98, Additional File 2 - Table S3). Overall, transcript dominance was less accentuated than in primary tissues - 69% (63.11% to 71.17%, SD = 2.40) of genes with a two-fold dominant major transcript and 42% (35.16% to 44.76%, SD = 2.90) with a five-fold dominant transcript in cytosol (Additional File 2 - Table S4). These differences could reflect higher transcription and splicing rates in cell lines, although they could also be due to technical variability between the two datasets.

**Figure 2:**
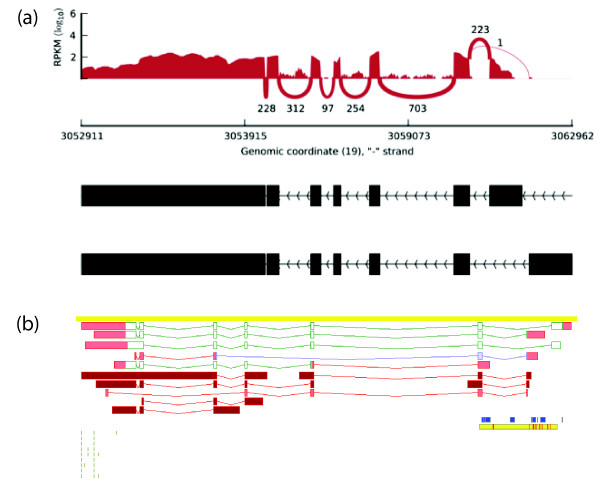
**Example of non-canonical major transcript common to all the 16 tissues analysed: AES (amino-terminal enhancer of split, ENSG00000104964)**. Read coverage for the gene (**a**) and screenshot from the Zmap manual annotation interface (**b**). UTR exons and splice variants with no annotated CDS are shown in red, coding exons are shown in green and the CDS portion of models annotated as NMD are shown in purple. Clusters containing >8,000 CAGE tags defining transcription start suites are shown as small blue boxes, CpG islands are shown as yellow boxes broken by horizontal red bars representing TSS predictions from EPONINE [59]. The short horizontal green bars represent polyadenylation sites identified by polyAseq [60].

Given that estimating transcript expression from short reads is a challenging task, we performed additional analyses to test the reliability of our observations. First, we simulated different RNA-seq datasets to test whether the method used can distinguish between two hypothesised scenarios - one dominant transcript per gene *vs*. similar expression levels of the different transcripts in each gene (see Methods) - and concluded that our method reliably discriminates between those, even when taking into account different sequencing depths (Additional File 1 - Figure S6). In addition, this analysis reveals that the method is not biased towards the identification of a single transcript per gene (see Additional File 1 - Figure S6) and reproduces our previous findings about transcript dominance (Additional File 2 - Table S7). Second, we used alternative methods to estimate transcript expression levels and identify major transcripts, including direct evidence from junction reads. All the methods have a strong overlap in the predictions (see Additional File 2 - Table S8 and Methods). Third, we analysed the length distribution of major transcripts to determine whether the identification of major transcripts is biased towards the longest one for each gene. We observe that the length of the major transcript is distributed widely (Additional File 1 - Figure S7) and that in >50% of the cases (50.98% to 55.46%, SD = 1.53) the major transcript is not the longest one annotated (Additional File 2 - Table S9 and Additional File 3 - File S1). The same trend is observed when taking into account CDS length: we estimate that in approximately 50% of the cases (44.42% to 48.23%, SD = 1.12) the major transcript does not contain the longest CDS, thus not corresponding to the 'canonical' transcript as annotated in UniProt (Additional File 2 - Table S9 and Additional File 3 - File S1). For instance, we identified the *AES *(amino-terminal enhancer of split) and *CD47 *(CD47 molecule) genes, for which we detect a ubiquitous major transcript that is shorter than the current reference (Figure 2 and Additional File 1 - Figure S8). Finally, we addressed the impact of unnanotated transcripts in our observations by performing *de novo *transcript identification using Cufflinks (see Methods). As expected, we observe a higher number of transcripts per gene (6.38 in GENCODE v11 *vs*. 12.84 using Cufflinks), although the main message still prevails (Additional File 1 - Figure S9 and Additional File 2 - Table S10).

### Major transcripts tend to be recurrent across samples

**Figure 3:**
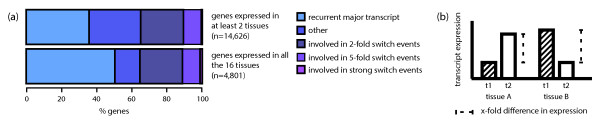
***Expression patterns for major transcripts**. (**a**) Percentage of genes with recurrent and non-recurrent major transcripts. Changes in the identity of major transcripts across samples were quantified with switch events*. (**b**) Concept of switch event. A gene is considered to be involved in a switch event if we detect two different dominant major transcripts in two different samples. If the dominant transcripts involved in the switch are expressed above 5 FPKM, while the minor ones are expressed below 1 FPKM, we define the event as a strong switch.

We next sought to quantify how often we detect the same major transcript across different samples. Focusing on the tissue dataset, and taking into account genes that are expressed in at least two different tissues, we estimate that this is the case for 35% of the genes (Figure 3a and Additional File 3 - File S2); while approximately 50% of the genes that are ubiquitously expressed (that is, expressed in all the 16 tissues) have the same major transcript (Figure 3a). For higher expression thresholds the overlap in the major transcript increases to 79% (Additional File 2 - Table S11). In addition, comparison of expression patterns for major and minor transcripts revealed that the former tend to be expressed in a recurrent fashion (Additional File 1 - Figure S10). In the cell line dataset, we detected similar major transcript expression patterns, with substantial differences depending on the subcellular fraction analysed (see Additional File 4 - Supplementary Results).

**Figure 4:**
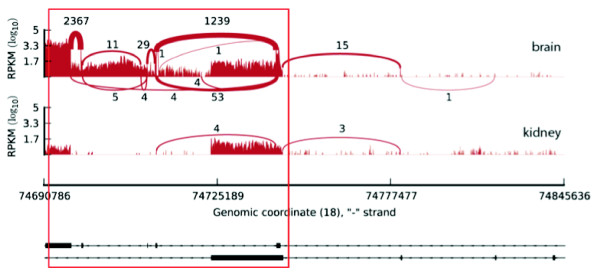
**Example of a switch event: *MBP *(myelin basic protein, ENSG00000197971)**. Read coverage for the gene in brain and kidney. Further tissues, as well as transcript annotation information, can be visualised in Additional File 1 - Figure S12.

On the other hand, we still detect a significant fraction of genes (>60%) for which the identity of major transcripts changes across conditions. To quantify these differences, we study switch events - changes of dominant transcripts between samples. We define a gene to undergo a two-fold (or five-fold) switch between two samples, if this gene has two different two-fold (or five-fold) dominant transcripts in the respective samples, while the overall expression of the gene does not change abruptly between the two samples (see Figure 3b and Methods). From the pairwise comparison of the 16 tissues, we found that approximately 35% of the studied genes (*n *= 14,626) are involved in two-fold switch events, and approximately 10% in five-fold switch events. However if we additionally require the dominant transcripts to be expressed over 5 FPKM and the minor ones under 1 FPKM, thus increasing the chance that the switch might have an effect at the protein level, the number of such strong switches drops to <1% (Figure 3a, Additional File 2 - Table S12 and Additional File 3 - File S4). Further focusing on strong switch events across tissues, we detected only 67 genes for which the switch implied a change in the protein sequence. Transcript expression profiles for this last subset of events can be visualised in Additional File 1 - Figure S11. One example is the *MBP *gene (Myelin Basic Protein), for which we detect a brain specific major transcript (Figure 4 and Additional File 1 - Figure S12).

As our tissue dataset lacks biological replicates, we cannot distinguish which of the switch events are tissue specific, as opposed to individual specific or due to technical or biological noise. Reassuringly, we observed that genes with high variability in splicing across tissues are enriched for transit peptides, among other GO terms (Additional File 2 - Table S13 and Methods), thus suggesting that biological variability dominates above technical variability. In addition, in order to estimate to what extent technical variability could influence our estimates, we repeated the same analyses in the cell line dataset, where we observed that the number of switch events detected across cell lines is significantly higher than the one detected across replicates (that is, 10 times higher for five-fold switch events, Additional File 1 - Figure S13). Overall, the proportion of switch events detected in the cell line data is lower than the one observed in the tissue data (Additional File 2 - Table S12 and Additional File 3 - File S5).

### Major transcripts do not always code for proteins

**Figure 5:**
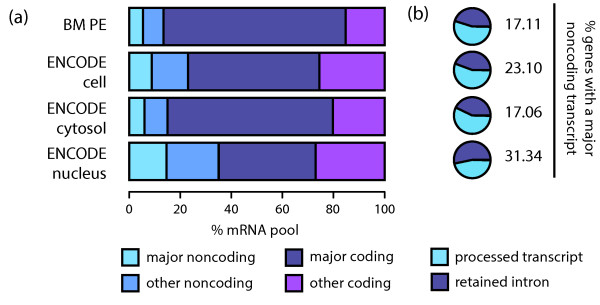
**Major non-coding transcripts in protein coding genes**. (**a**) *Proportion of the mRNA studied represented by different categories of transcripts. Average proportions were calculated including all the samples from each dataset. Major non*-*coding transcripts are more abundant in nucleus, where the proportion of major coding ones also becomes reduced*. (**b**) Transcript biotype categories for the major non-coding transcripts. Average proportions were calculated including all the samples from each dataset. Processed transcripts are more abundant in the cytosol, while retained introns represent the major fraction in the nucleus. Other minor categories that represented <1% of the transcripts were also identified, but are not visible in the plots.

Functional classification of major transcripts revealed that for 17% (15.26% to 20.64%, SD = 1.60) of protein coding genes expressed in primary tissues the major transcript lacks an annotated CDS as indicated by GENCODE (see Additional File 4 - Supplementary Methods). Taking into account expression levels, and focusing on cell line data, we observe that major non-coding transcripts are more abundant in the nucleus, where they represent approximately 15% of the studied mRNA pool (12.99% to 16.66%, SD = 1.10, Figure 5a). Genes with major non-coding transcripts are expressed at higher levels in the nucleus, compared to those with major coding transcripts, while this trend is inverted in the cytosol (Additional File 1 - Figure S14). In addition, non-coding major transcripts are less dominant than coding ones in both compartments (Additional File 1 - Figure S14): 61% (54.80% to 65.57%, SD = 3.07) of major coding transcripts are also five-fold dominant, while this number goes down to 27% (15.71% to 35.34%, SD = 5.76) for non-coding major ones. Finally, the annotation revealed that the major non-coding transcripts correspond to retained introns and processed transcripts, which lack an open reading frame (see Supp. Methods). We observe a higher proportion of processed transcripts in the cytosol and retained introns in the nucleus (Figure 5b).

In order to evaluate the hypothesis that incomplete splicing could explain the higher proportion of major retained introns in the nucleus, we compared intron expression levels across cellular compartments (see Methods for details on the calculation of intron expression). We observe slightly higher intron expression in the nucleus compared to the cytosol (Additional File 1 - Figure S15). We also observe a general trend in the location of major retained introns towards the transcriptional 3'-end (Additional File 1 - Figure S15); moreover this trend is more accentuated in the cytosol than in the nucleus, where it is possibly masked by the higher intronic expression levels. Such 3' intron retention has been previously linked to nonsense-mediated decay (see Discussion). Alternatively, the prevalence of retained introns as a major transcript could point to a functional mechanism. We observe that genes with retained introns as the major transcript both in nucleus and cytosol are expressed at lower levels in the later (Additional File 1 - Figure S15), which would be consistent with a regulatory role for retained introns (see Discussion). We also detect that those genes are associated to transit peptides and ribosomal components, which is consistent with previous findings indicating that introns regulate the expression of ribosomal proteins in yeast (Additional File 2 - Table S14, see Discussion). On the other hand, the term 'processed transcript' constitutes an ambiguous category. Manual inspection of a subset of processed transcripts that were consistently identified across all samples as the major transcript suggests that potentially they could be re-annotated to protein coding, nonsense mediated decay (NMD) or retained intron (Additional File 2 - Table S15). Together, this seems to suggest that the true proportion of non-coding major transcripts for protein coding genes may be lower than the current annotation suggests, and most of these result from retained introns, which can be explained by incomplete splicing or potentially have a regulatory role.

## Conclusions

In this study we combine RNA-seq data from different primary tissues, cell lines and cellular compartments to characterise the human protein coding transcriptome from a functional perspective. We show that in a given condition most protein coding genes not only express one major transcript, as recently observed by Djebali *et al. *[13], but in most cases the major transcripts are dominating the transcriptome. This observation is accentuated when grouping transcripts by TSS, a scenario in which differences in transcript abundance can be mostly attributed to splicing. We are aware that transcript quantification from short read sequences is not a trivial task, and that the current annotation is continuously updated to include novel transcripts. However, our findings are supported by several quantification methods, including *de novo *transcript discovery, they are consistent across all datasets, and are reassuringly supported by direct evidence from junction reads. In addition, the single transcript dominance becomes stronger for highly expressed genes, for which transcript prediction and quantification have been reported to be more reliable (RNA-seq Genome Annotation Assessment Project - RGASP, J Harrow, T Steijger, F Kokocinski, JF Abril, C Howald, A Reymond, A Mortazavi, B Wold, T Gingeras, R Guigó, *et al*., in preparation). In the long term, longer reads and single cell sequencing will shed more light on the topic.

Changes in alternative splicing across conditions have been widely reported, with many studies focusing on the differences across tissues ([7,8,20], Merkin *et al. Science *2012, [24]), where splicing is thought to control the interactions of the protein products [25,26]. Here we quantify changes in the major transcript across conditions by looking at switch events. We detect a significant number of genes that express several major transcripts across different conditions. Relevant examples include the *MBP *gene, *PSEN1 *(presenilin 1) and *ILF3 *(interleukin enhancer binding factor 3). However, in many cases the differences are subtle. This would suggest that alternative splicing might be more prevalent in dynamic processes such as development and differentiation, rather than steady state. On the other hand, dominant transcripts that are recurrent in many samples are also interesting, given that they can be used to build a catalogue for the reference transcriptome. A closer inspection of a set of recurrent major transcripts revealed cases where they do not contain the longest CDS, a criteria often used in resources like UniProt to define a reference transcript [27], thus exposing some of the limitations of the current definitions and pointing to potential advantages of taking into account functional data. For instance, the major transcripts detected for the *AES *gene and the *CD47 *gene are ubiquitously expressed and do not correspond to the current reference.

We were surprised to find that for a non-negligible fraction of protein coding genes the major transcript is non-coding and can be classified as retained intron or processed transcript, which lack an open reading frame [28]. However, we observe higher prevalence of non-coding major transcripts in the nucleus, specifically retained introns. Evidence exists suggesting that unspliced or incompletely spliced mRNAs are confined to the nucleus [29,30]; therefore we hypothesize that our observation could reflect incomplete splicing, as suggested by the higher expression levels of introns in nucleus. We also observe that retained introns are preferentially located towards the transcriptional end of transcripts, which has been previously linked to nonsense mediated decay [31], a control mechanism that leads to the degradation of unspliced transcripts when they are transported to the cytosol [32,33]. Nevertheless, several cases of functionally relevant retained introns have been described, either as a mechanism to target mRNA molecules (for example, [34,35], produce alternate protein products [36] or regulate expression levels [37-39]. We observe that genes with retained introns as the major transcript in both nucleus and cytosol are expressed at considerably lower levels in the later, which could point to a regulatory role. Finally, we also detected that those genes are associated to ribosomal components, which is consistent with previous findings indicating that introns regulate the expression of ribosomal proteins in yeast [40]. On the other hand, we were able to re-annotate some of the recurrent processed transcripts to either coding or retained intron, thus illustrating a potential application of our analyses. For example, we revisited the annotation for one of the transcripts from *PARK7 *(parkinson protein 7) to protein coding, from *RNF40 *(ring finger protein 40) to nonsense mediated decay and from *OSBPL2 *(oxysterol binding protein-like 2) to retained intron.

Overall, it is difficult to predict the impact of our observations at the protein level. There have been several studies addressing the relationship between protein and transcript levels, which in general point at a modest, but not insignificant correlation [17,21,41,42]. Translational efficiency, mRNA and protein turnover rates are likely to have an impact on protein levels [21]. On the other hand, proteomics studies also show that observing a protein is unlikely unless there are at least a certain number of RNA molecules per cell [21-23]. This may be partly due to insufficient sensitivity of the methods used; nevertheless this supports the hypothesis that the abundance of proteins corresponding to minor transcripts is likely to be lower than the one corresponding to dominant transcripts. We evaluated this hypothesis by overlapping our set of dominant transcripts with a set of transcripts predicted to be translated into proteins by entirely independent means [43]. We detected a considerably larger overlap for those transcripts that have a tendency to be identified as major, compared to minor ones, suggesting that major transcripts could be preferentially translated. On the other hand, alternative splicing not only has an impact on the proteome repertoire [44-46], but also at the transcriptome level, cooperating in the control of expression levels [20,32,47] and contributing to spatial expression patterns through transcript localisation [46]. This brings in other potential roles for minor transcripts. However, it is also possible that some of those minor transcripts are simply the result from noisy splicing [22,45,48].

The discovery of alternative splicing and many different classes of non-coding RNAs, together with the establishment of RNA-seq, revealed that the number of transcripts exceeds many times the number of genes in the human genome. This has been used to argue that alternative splicing possibly explains the low number of genes compared to what was believed before it was sequenced [45]. Despite this diversity of transcripts, our findings indicate that most protein coding genes express one dominant transcript in a given condition and that most of the mRNA pool from protein coding loci arises from major transcripts, thus suggesting that those could be the main contributors to the proteome. In addition, the ratio of the number of expressed transcripts to genes in primary tissues is on average 1.12 and many of the dominant transcripts are the same across different conditions. Overall, these observations may help proteome analysis by prioritising the candidate proteins that are more likely to be present in a given sample. At the same time, identification of changes in the major transcript across conditions can lead to relevant clinical findings (for example, [18]) and may also be used to predict the functional impact of the detected changes in variation studies.

## Materials and methods

### Datasets and mapping

We based our analyses on the Illumina Body Map (BM) dataset and a subset of ENCODE cell lines [49] (ArrayExpress accession ids: E-MTAB-513 and E-GEOD-26284, respectively), jointly covering a total of 21 different tissues and cell lines, as well as different cellular compartments. Raw fastq files were retrieved from the European Nucleotide Archive [50] using the accession numbers indicated in Additional File 2 - Table S16. In addition to the publicly available datasets, we simulated two RNA-seq experiments using the Flux Simulator [16]. Details on the parameters used in the simulation have also been listed in Additional File 2 - Table S16.

Fastq files in the BM dataset were filtered before mapping by trimming the last five nucleotides of all reads. Raw data were mapped to the human genome and transcriptome (Ensembl 66; [1]) using TopHat v1.3.3 [51] and Bowtie v0.12.7 [52], respectively.

### Gene and transcript study sets

Gene and transcript annotations used in the analyses correspond to those in GENCODE v11 [28]. We focused on protein coding genes and filtered out those for which at least one of the annotated transcripts was shorter than 300 bp, given that those transcripts would be lost during the size selection step in the RNA-seq experiment. In total, our study set comprises 18,450 protein coding genes, of which 14,902 have more than one transcript annotated.

### Counting reads overlapping exonic and intronic regions

Exonic coordinates were retrieved from the annotation and used to define intronic regions. Formally, our definition of intron encompasses those regions that are located inside genic boundaries and are not overlapped by any exon in any annotated transcript. We then computed the number of reads overlapping known exons and introns using dexseq-count (DEXSeq v1.5.5 [53]) and converted read counts to FPKM values with custom scripts.

### Estimation of gene and transcript expression levels

For each gene, expression levels were calculated as the average FPKMs of all expressed exons. Independently, transcript abundances were obtained using three different tools: MISO v0.4.1 [6], Cufflinks v1.3.0 [4] and MMSEQ v0.10.0 [5]. MISO and Cufflinks take as input alignments to the genome, while MMSEQ requires mapping to the transcriptome, thus the need to use two different mapping strategies (see above). In all three cases we based the estimates on the existing transcript annotation (see above), cancelling any option for *de novo *inference, and converted those to transcript relative abundances when necessary. In this manuscript we are referring to the results obtained by MISO and we use a default FPKM threshold of 1 to consider a gene/transcript as expressed. This threshold has been suggested as the minimum expression required for protein detection [21-23] and is different from lower thresholds that have been suggested to address transcript detectability [54]. In addition, we include higher expression thresholds in the analyses (5 and 10 FPKM), since transcript quantification has been reported to be more reliable for those (RNA-seq Genome Annotation Assessment Project - RGASP, J Harrow, T Steijger, F Kokocinski, JF Abril, C Howald, A Reymond, A Mortazavi, B Wold, T Gingeras, R Guigó, *et al*., in preparation). Finally, we consider a transcript as detected independently of its expression level, given that the gene is expressed.

mRNA pool estimates were calculated as introduced by [54]. Briefly, the fraction of the studied mRNA pool that can be explained by the expression of major transcripts can be represented as the ratio of the sum of FPKMs for major transcripts *vs*. the sum of FPKMs for all the transcripts in our study set. All transcripts encoded within protein coding genes were taken into account in the calculation, independently of their transcript biotype, and thus we refer to mRNA pool from protein coding loci. Mitochondrial genes in our study set were discarded for this analysis (*n *= 11 in our study set), since they are present multiple times in the cell and could bias the quantification (for example, we found that six transcripts arising from mitochondrial genes explain almost 50% of the studied mRNA pool).

### Direct evidence from junction reads

Starting with our gene study set (see above), we focused on those genes for which all of the annotated transcripts can be uniquely identified by at least one splice junction (*n *= 2,306). We then proceeded to identify major transcripts based on coverage evidence (that is, quantifying the number of reads supporting each junction and taking the average in case of several splice junctions). For each sample we calculated the overlap with MISO (Additional File 2 - Table S8).

### *De novo *transcript discovery using Cufflinks

We used Cufflinks v1.3.0 [4] to discover novel transcripts in each tissue from the BM dataset and merged all the obtained annotations using cuffmerge. We then focused on the subset of transcripts that overlap with known protein coding genes and filtered out those genes with transcripts shorter than 300 bp, as mentioned previously (see Gene and transcript study sets). A summary on the number of genes and transcripts identified can be found in Additional File 2 - Table S10.

### GO analysis

GO analyses were performed with the DAVID software [55,56]. The reference population was defined by our gene study set (see above) and an adjusted *P *value of 0.05 (Benjamini and Hochberg correction [57]) was used as a threshold for the identification of significant GO terms.

### Switch events

We performed a pairwise comparison of the samples in each dataset and focused on those cases where we detect different major coding transcripts. Given a gene *G*, a pair of transcripts *I_k _*and *I_l _*and a pair of samples *S_i _*and *S_j_*, we say that gene *G *undergoes an x-fold switch between transcripts *I_k _*and *I_l _*in samples *S_i _*and *S_j_*, if *G *is expressed in *S_i _*and *S_j _*and the ratio of the expression of *I_i _*to the expression of *I_j _*is at least *x *in *S_i _*and no more than *1/x *in *S_j_*. Additionally, we looked at x-fold switch events that are not accompanied by strong change in the overall gene expression, to filter out the cases where the change is largely due to the overall expression change. A switch event was considered to be expression dependent if the difference in the expression level of gene *G *between sample *S_i _*and *S_j _*was bigger than the mean, and expression independent otherwise. Finally, we say that there is a strong switch if the expression of *I_k _*in *S_i _*and *I_l _*in *S_j _*is at least 5 FPKM, while *I_k _*in *S_j _*and *I_l _*in *S_i _*less than 1. The intuition behind the definition of strong switch is that we want to maximise the chances of obtaining a protein in the first sample and not in the second, and *vice versa*, and several proteomics studies show that observing a protein is unlikely unless there are at least a certain number of RNA molecules per cell [21-23]. Finally, given a sample S and a switch (I_k_, I_l_) we can calculate ratio r = expression(I_k_)/expression(I_l_) and its logarithm lr = log(r). Given a switch (I_k_, I_l_) and set of samples S_1_, ..., S_j_, the vector (lr_1_, ..., lr_j_) of these values is called the expression profile of the switch.

### Variability in splicing across tissues

Variability in splicing relative abundances across tissues was measured using the method introduced by [58].

## Abbreviations

BM: Illumina Body Map dataset.

## Authors' contributions

MGP carried out the analyses and wrote the manuscript. AF and JR participated in the analysis and writing. JH participated in the design and writing. AB conceived the study and wrote the manuscript. All authors read and approved the final manuscript.

## Supplementary Material

Additional file 1 Supplementary Figures Click here for file

Additional file 2 Supplementary Tables Click here for file

Additional file 3 Supplementary Files Click here for file

Additional file 4 Supplementary Methods and Results Click here for file
