# Supplementary material for: Transcriptome analysis of human tissues and cell lines reveals one dominant transcript per gene
Source: Genome Biol. 2013 Jul 1;14(7):R70. doi: 10.1186/gb-2013-14-7-r70 (PMC4053754; doi:10.1186/gb-2013-14-7-r70)
Supplement: Additional file 1 — Supplementary Figures [file gb-2013-14-7-r70-S1.PDF]

Supplementary Figures

Figure S1: Relative abundance of the subset of transcripts in each position of the ranking for all datasets, including different expression thresholds. Only genes with more than one transcript annotated are represented here.

Figure S2: Relative abundance of the subset of transcripts in each position of the ranking for the BM dataset, separating genes by the number of annotated transcripts.

Figure S3: Relative abundance of the subset of transcripts in each position of the ranking for all datasets, including different expression thresholds, when grouping transcripts by TSS. Only TSS with more than one transcript annotated are included here.

Figure S4: Ratio of expression levels between the major transcript and the second most abundant one for all datasets, relative to the number of annotated transcripts and including different expression thresholds.

Figure S5: Proportion of dominant vs non-dominant major transcripts detected in the BM dataset for different gene expression thresholds and taking into account different dominance degrees.

Figure S6: Relative abundance of the subset of transcripts in each position of the ranking for the simulated datasets, including different sequencing depths. Major transcript abundance is underestimated by the analysis pipeline used here, thus suggesting that it is not biased towards the identification of a single transcript per gene.

Figure S7: Length distribution for major transcripts, relative to the longest transcript annotated for each gene.

Figure S8: Example of a non-canonical major transcript: *CD47* (CD47 molecule, ENSG00000196776). Read coverage for the gene (a) and screenshot from the Zmap manual annotation interface. UTR exons and splice variants with no annotated CDS are shown in red, coding exons are shown in green and the CDS portion of models annotated as NMD are shown in purple. Horizontal pink bars represent manually annotated polyadenylation signals and adjacent black bars manually annotation polyadenylation sites. Clusters containing >8000 CAGE tags defining transcription start suites are shown as small blue boxes, CpG islands are shown as yellow boxes broken by horizontal red bars representing TSS predictions from EPONINE [59]. Pale blue boxes represent BLAST alignments of Swissprot proteins. The short horizontal green bars represent polyadenylation sites identified by polyAseq [60].

Figure S9: Summary of Cufflinks *de novo* quantification results for the tissue dataset. (a) Relative abundance of the subset of transcripts in each position of the ranking. (b) Percentage of the studied mRNA pool explained by each category of transcripts. (c) Expression distribution for major and minor transcripts. (d) Relative abundance of the subset of transcripts in each position of the ranking, separating genes by the number of annotated transcripts. (e) Ratio of expression between the major transcript and the second most abundant one, relative to the number of annotated transcripts and including different expression thresholds.

Figure S10: Comparison of expression patterns for major and minor transcripts.

(a) Percentage of major and minor transcripts expressed in a given number of tissues (left). Proportion of major vs minor transcripts in each number of tissues (right). Major transcripts tend to be broadly expressed (Spearman correlation: 0.90, p-value<$2.2e^{-16}$).

(b) Recurrence degree of major and minor transcripts. Transcript breadth of expression (ie number of tissues in which a transcript was detected) relative to gene breadth of expression (ie number of tissues in which the corresponding gene was detected) was used as a recurrence measure. Compared to minor transcripts, major ones tend to be expressed in a recurrent fashion.

Figure S11: Expression profile for a subset of the identified switch events across tissues. Strong switch events with changes at the protein sequence level are represented here.

Figure S12: Read coverage for the *MBP* gene across the primary tissues analysed. Read coverage across the 16 tissues in the BM dataset (a) and screenshot from the Zmap manual annotation interface (b). UTR exons and splice variants with no annotated CDS are shown in red, coding exons are shown in green and the CDS portion of models annotated as NMD are shown in purple. Clusters containing >8,000 CAGE tags defining transcription start suites are shown as small blue boxes, CpG islands are shown as yellow boxes broken by horizontal red bars representing TSS predictions from EPONINE [59]. The short horizontal green bars represent polyadenylation sites identified by polyAseq [60].

Figure S13: Average number of genes involved in switch events in cell lines.

Figure S14: Focus on major noncoding transcripts. Protein coding genes for which the most abundant transcript is noncoding are expressed at higher levels in the nucleus, while the trend gets inverted in the cytosol (left). The difference in expression between the first and second most abundant transcripts becomes reduced when the dominant transcript is noncoding and in the nucleus in general (right).

Figure S15: Focus on major retained introns.

(a) Normalised intron expression in different cellular compartments. FPKMs were calculated for all the introns and normalised by gene expression levels (see Methods). Intron expression is higher in the nucleus than in the cytosol (Wilcoxon test p-value < $2.2e^{-16}$).
(b) Location of the dominant retained introns within the context of protein coding transcripts. Genes for which the most abundant transcript is a retained intron were initially considered in the analysis. Further filtering of this subset of genes included those for which the second most abundant transcript is protein coding and includes the dominant retained intron. Similar criteria were applied to analyse other less abundant retained introns. The location of the retained introns was then represented by measuring the distance from their centre to the transcriptional start of the selected coding transcript, as illustrated in the panel below the figure. Retained introns are preferentially located towards the transcriptional end of protein coding transcripts, and this trend becomes more apparent in the cytosol.
(c) Number of genes with a major retained intron in nucleus and cytosol.
(d) Expression levels in each cellular compartment for genes with a major retained intron both in nucleus and cytosol (n=81). Expression of this set of genes is higher in the nucleus (Wilcoxon test p-value < $2.2e^{-16}$).

Figure S16: Overlap in major transcript predictions within and across replicates for the ENCODE dataset. The detected overlap is higher when comparing replicates than across different cell lines (Wilcoxon test p-value = $2.514e^{-06}$).

SFIG 1

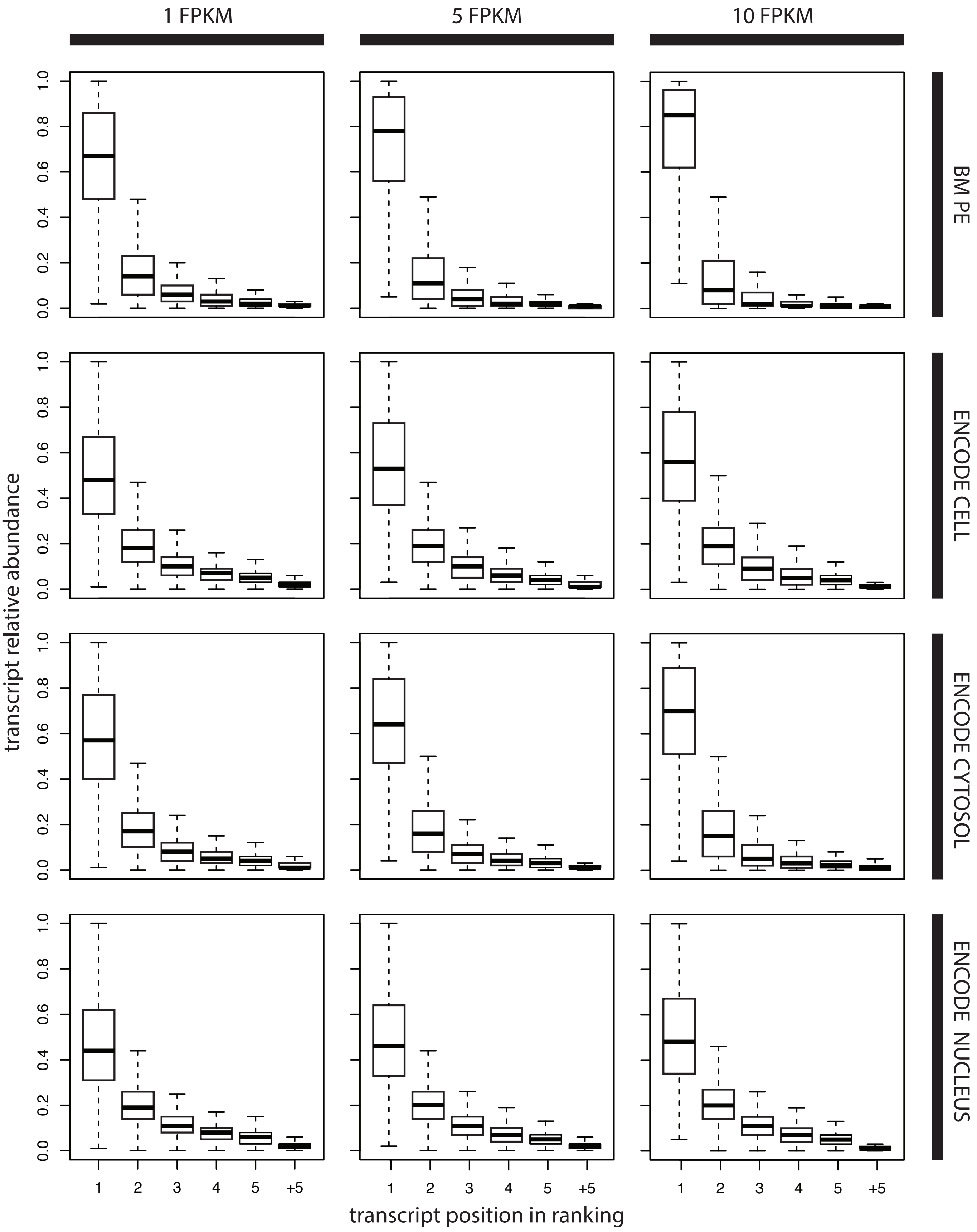
1 FPKM
5 FPKM
10 FPKM
BM PE
ENCODE CELL
ENCODE CYTOSOL
ENCODE NUCLEUS
transcript relative abundance
0.0
0.2
0.4
0.6
0.8
1.0
1
2
3
4
5
+5
transcript position in ranking

SFIG 2

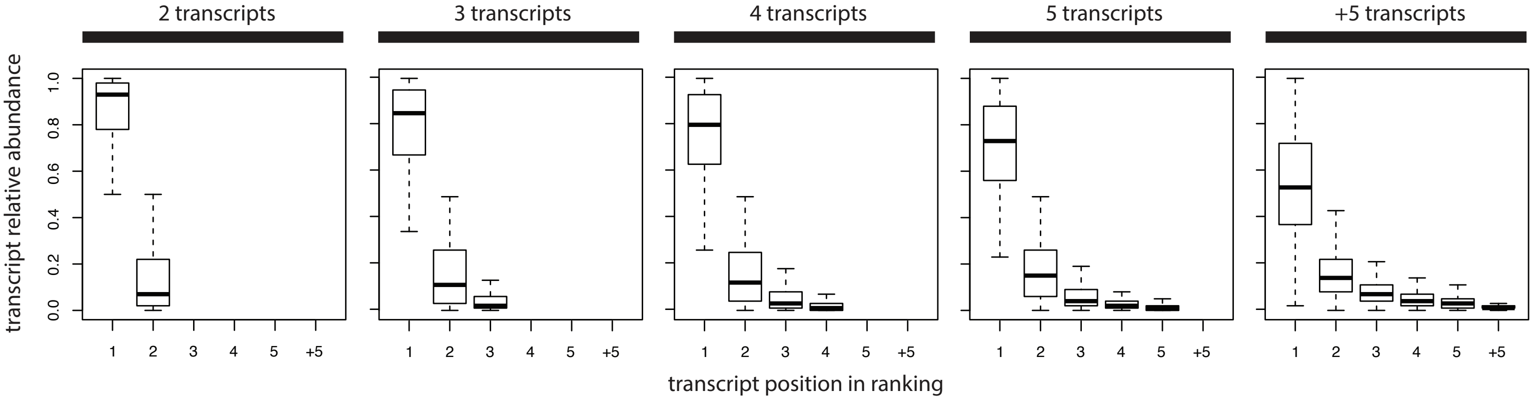
2 transcripts
3 transcripts
4 transcripts
5 transcripts
+5 transcripts
transcript relative abundance
0.0
0.2
0.4
0.6
0.8
1.0
1
2
3
4
5
+5
transcript position in ranking

SFIG 3

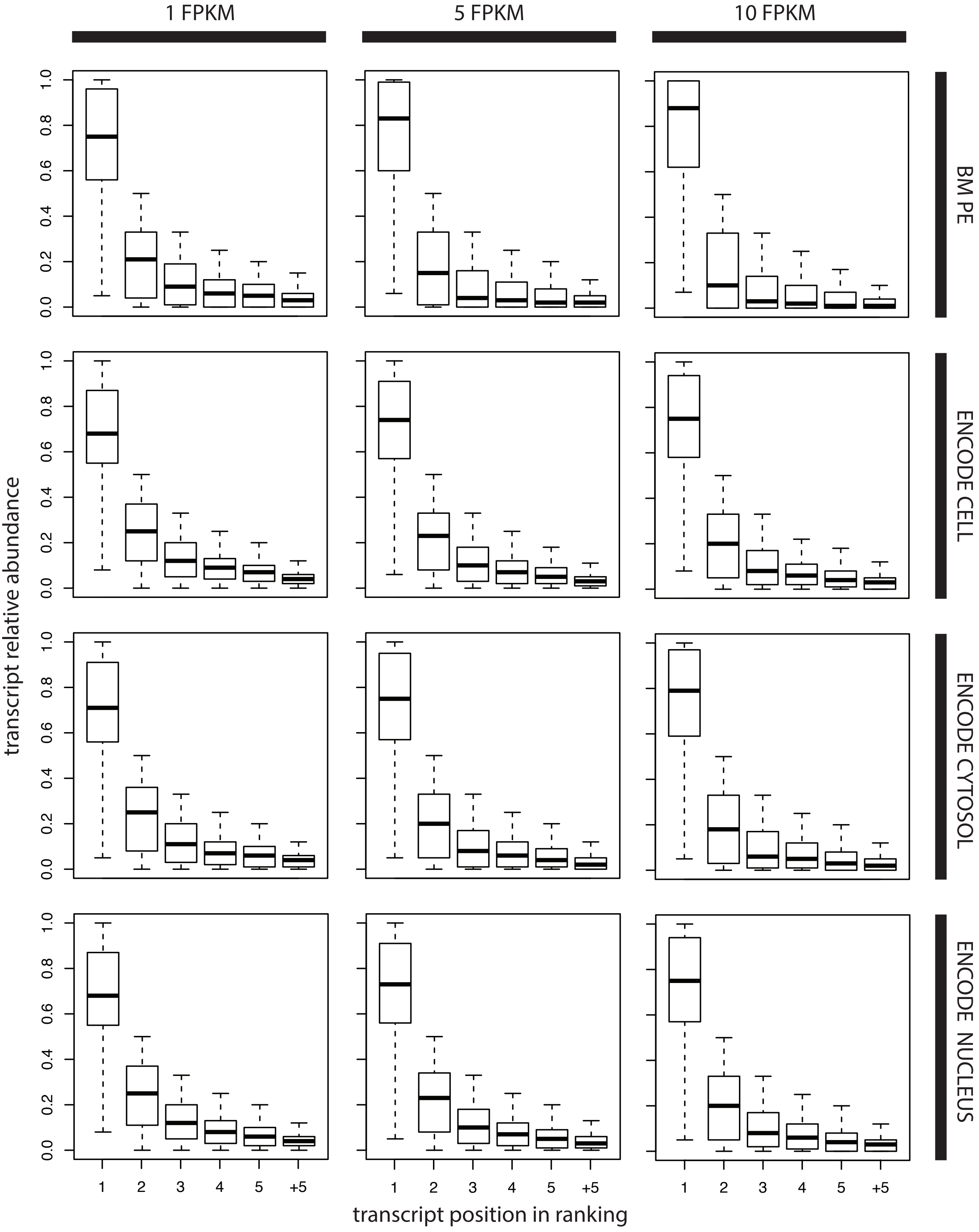

1 FPKM
5 FPKM
10 FPKM
BM PE
ENCODE CELL
ENCODE CYTOSOL
ENCODE NUCLEUS
transcript relative abundance
0.0
0.2
0.4
0.6
0.8
1.0
1
2
3
4
5
+5
transcript position in ranking

SFIG 4

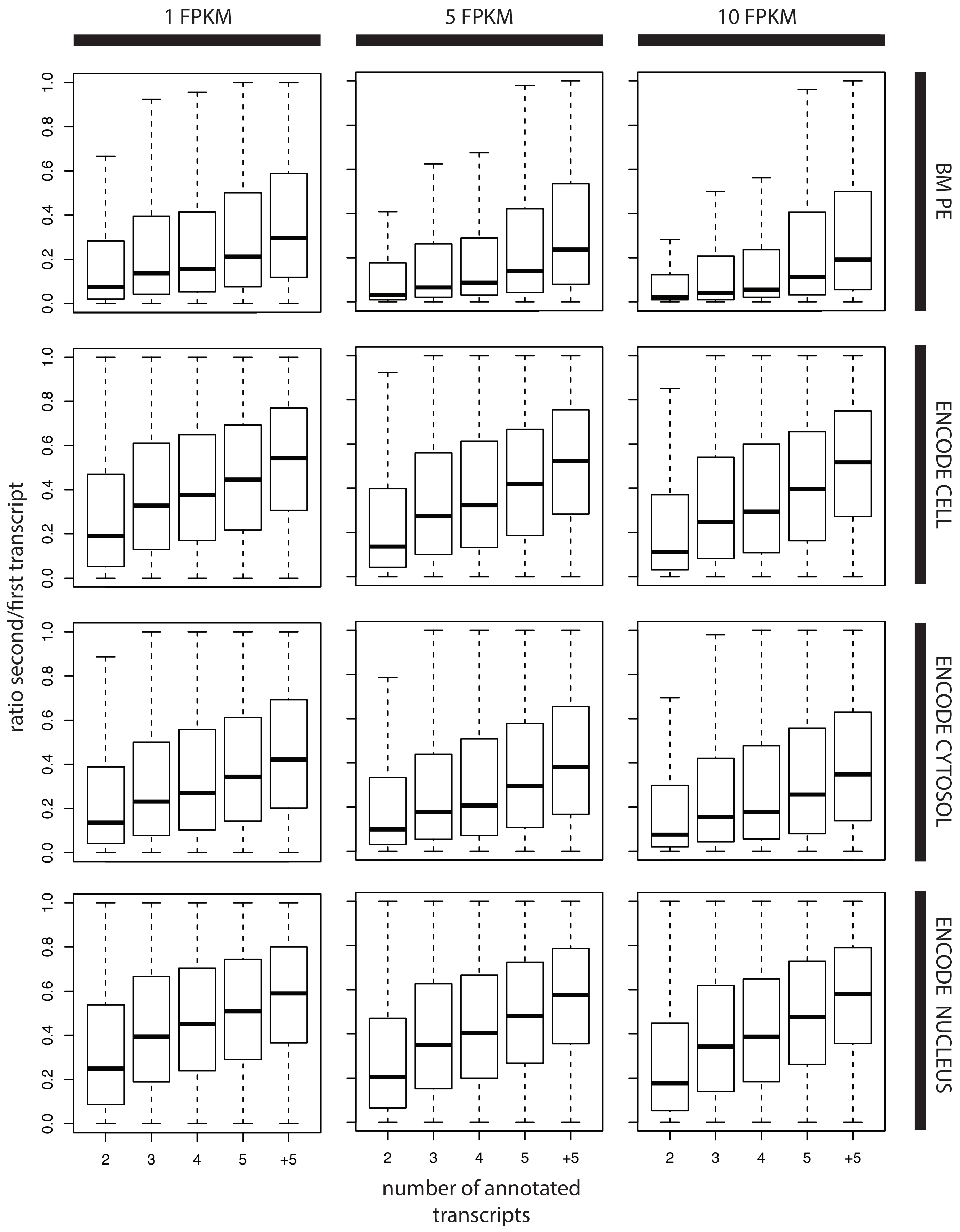
1 FPKM
5 FPKM
10 FPKM
BM PE
ENCODE CELL
ENCODE CYTOSOL
ENCODE NUCLEUS
ratio second/first transcript
0.0
0.2
0.4
0.6
0.8
1.0
2
3
4
5
+5
number of annotated transcripts

SFIG 5

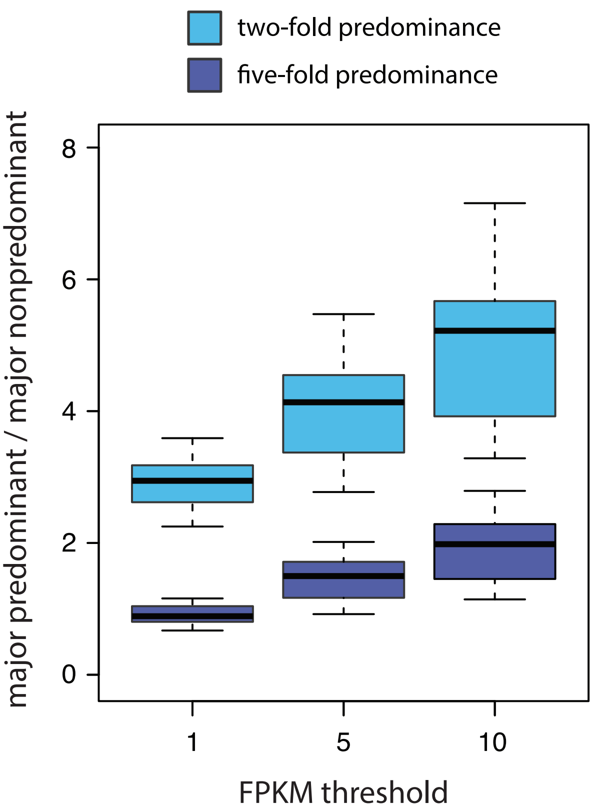

two-fold predominance
five-fold predominance
major predominant / major nonpredominant
0
2
4
6
8
1
5
10
FPKM threshold

SFIG 6

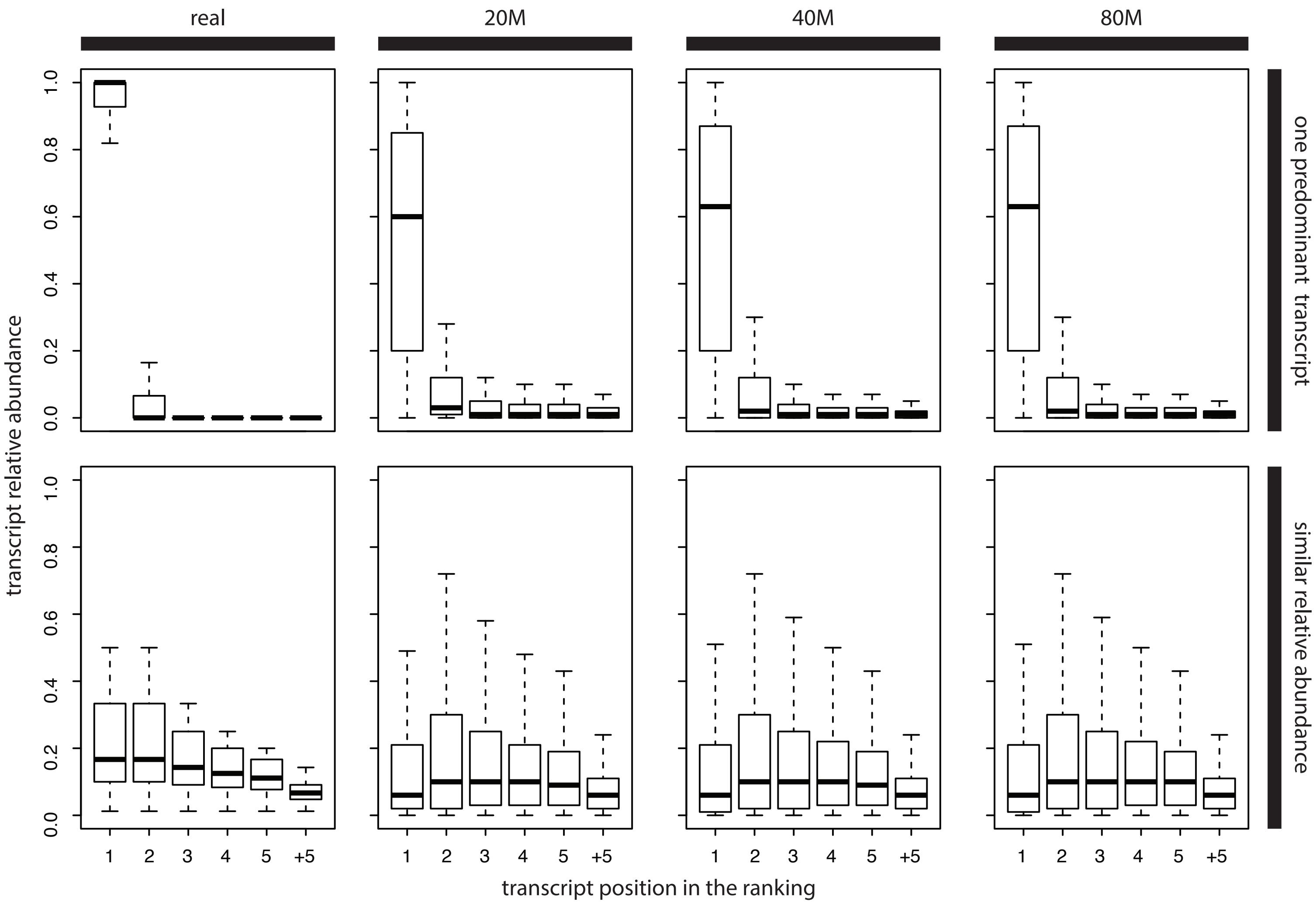

real
20M
40M
80M
one predominant transcript
similar relative abundance
transcript relative abundance
transcript position in the ranking
0.0
0.2
0.4
0.6
0.8
1.0
1
2
3
4
5
+5

SFIG 7

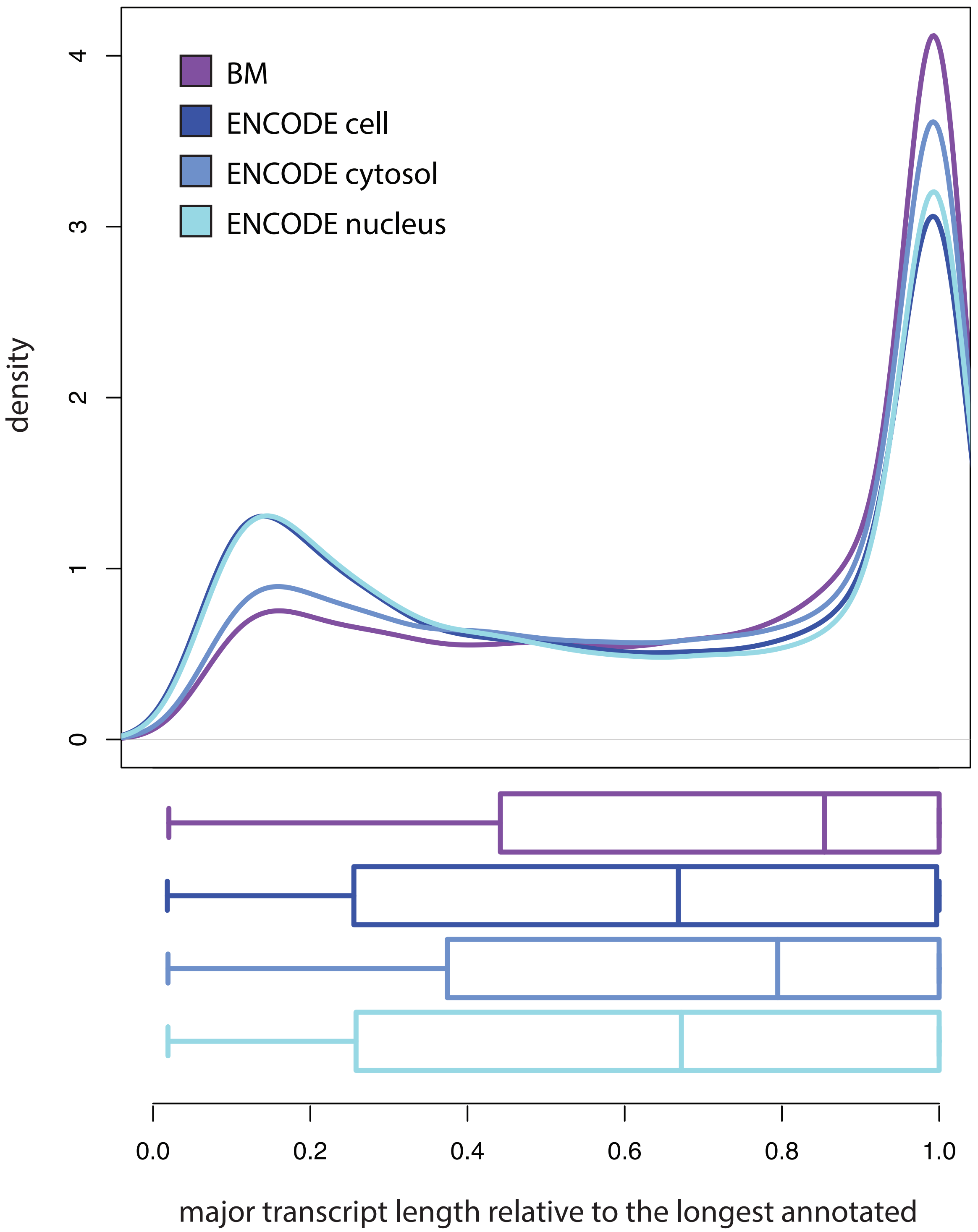
BM
ENCODE cell
ENCODE cytosol
ENCODE nucleus
density
0
1
2
3
4
0.0
0.2
0.4
0.6
0.8
1.0
major transcript length relative to the longest annotated

SFIG 8

(a)

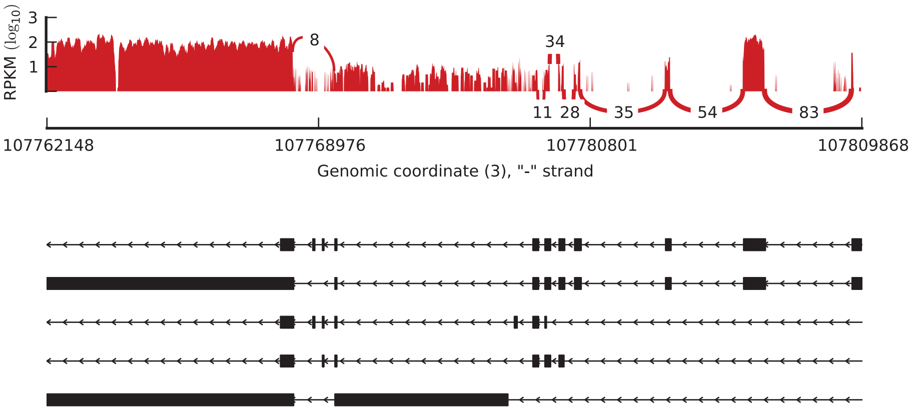

RPKM (log10)
3
2
1
8
34
11 28
35
54
83
107762148
107768976
107780801
107809868
Genomic coordinate (3), "-" strand

(b)

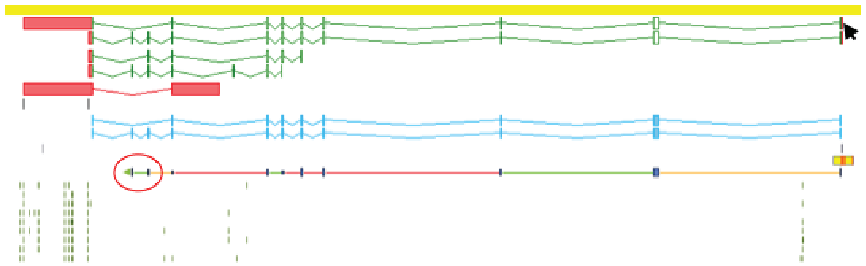

SFIG 9

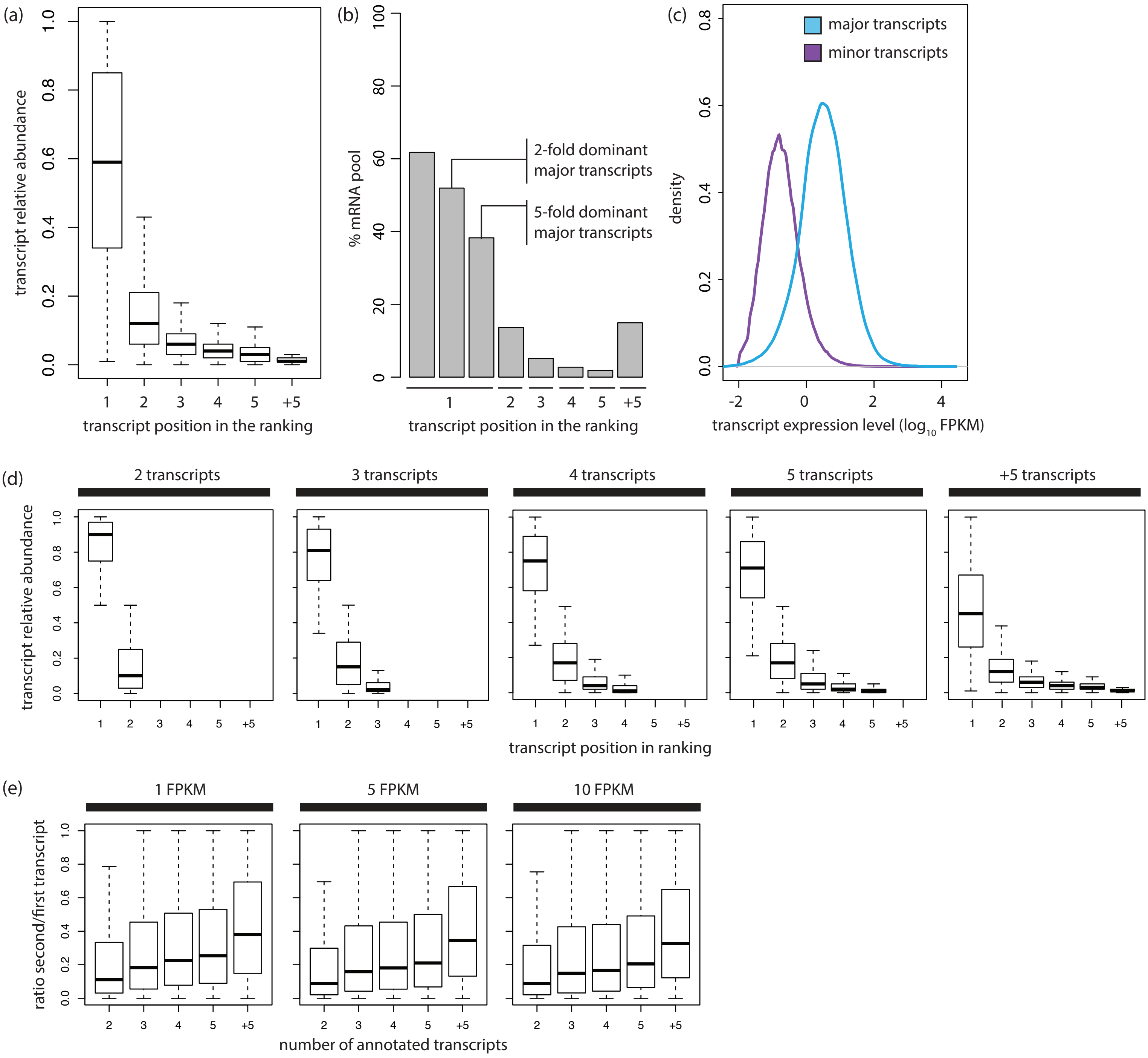

(a)
transcript relative abundance
1.0
0.8
0.6
0.4
0.2
0.0
1 2 3 4 5 +5
transcript position in the ranking
(b)
% mRNA pool
100
80
60
40
20
0
2-fold dominant major transcripts
5-fold dominant major transcripts
1 2 3 4 5 +5
transcript position in the ranking
(c)
density
0.8
0.6
0.4
0.2
0.0
major transcripts
minor transcripts
-2 0 2 4
transcript expression level (log_{10} FPKM)
(d)
2 transcripts
3 transcripts
4 transcripts
5 transcripts
+5 transcripts
transcript relative abundance
1 2 3 4 5 +5
transcript position in ranking
(e)
1 FPKM
5 FPKM
10 FPKM
ratio second/first transcript
2 3 4 5 +5
number of annotated transcripts

SFIG 10

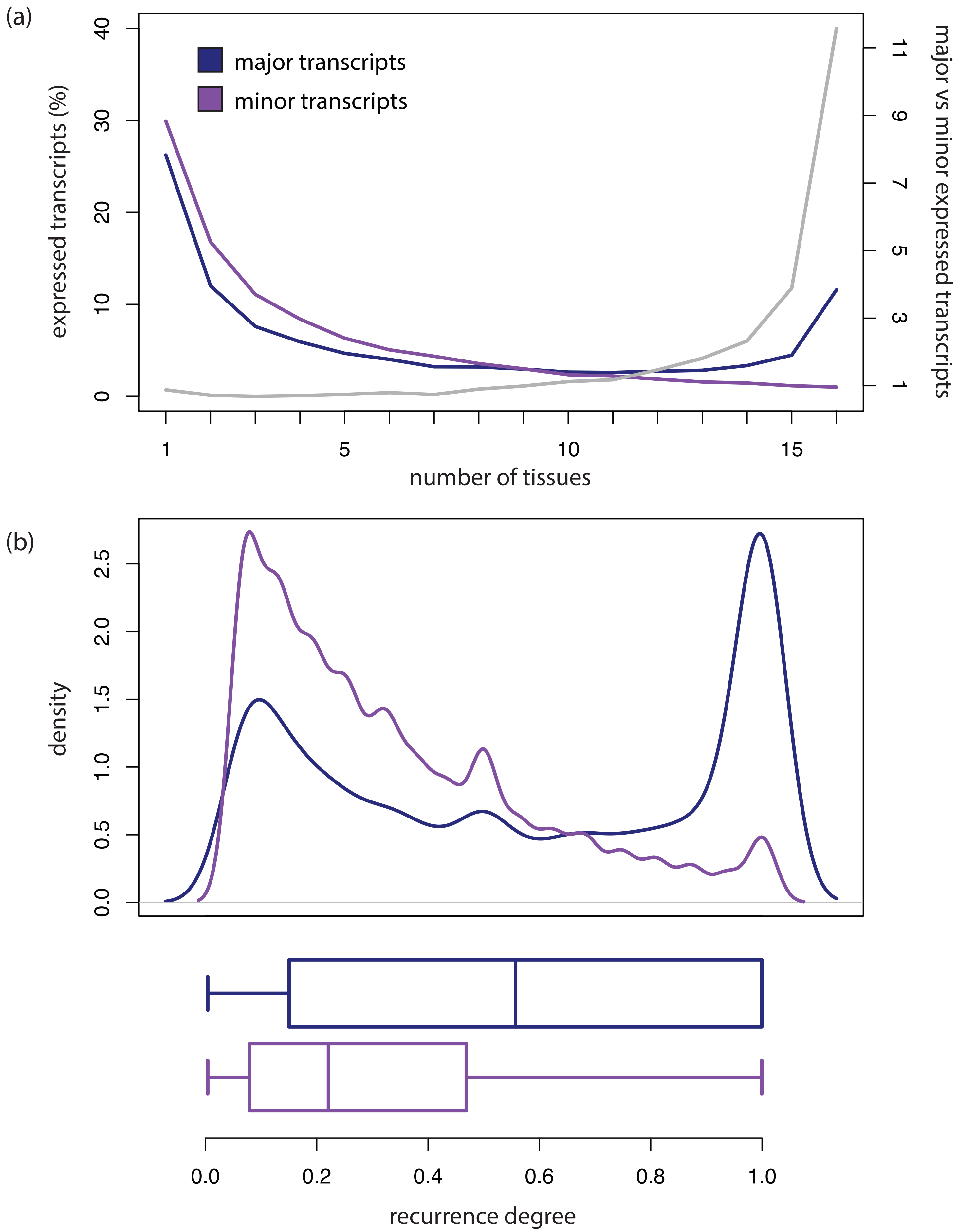

(a)
major transcripts
minor transcripts
expressed transcripts (%)
major vs minor expressed transcripts
number of tissues
0
10
20
30
40
1
3
5
7
9
11
1
5
10
15
(b)
density
0.0
0.5
1.0
1.5
2.0
2.5
0.0
0.2
0.4
0.6
0.8
1.0
recurrence degree

SFIG 11

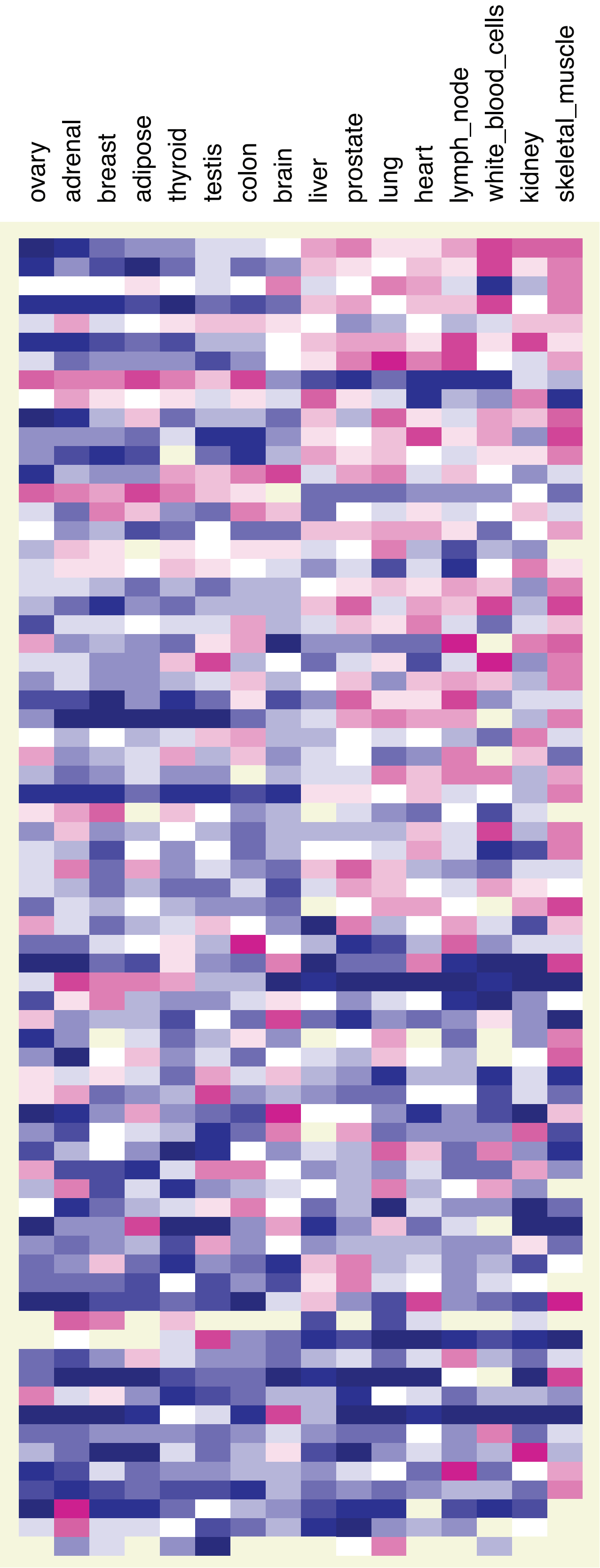

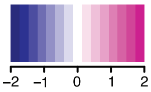

$$\text{expression profile} = \log_{10} \frac{\exp_A}{\exp_B}$$

transcript A	transcript B	
ENST00000356674	ENST00000409814	HNRNPA2B1
ENST00000368449	ENST00000448116	SHC1
ENST00000374199	ENST00000374198	PRPF4
ENST00000380381	ENST00000315377	RPS6
ENST00000399721	ENST00000327155	RBBP8
ENST00000569760	ENST00000474990	FUS
ENST00000223095	ENST00000445463	SERPINE1
ENST00000252440	ENST00000270517	ECSIT
ENST00000441391	ENST00000269346	TTYH2
ENST00000444226	ENST00000350766	MAPKAP1
ENST00000263270	ENST00000352203	AP2S1
ENST00000373053	ENST00000357157	MCU
ENST00000449870	ENST00000407004	ILF3
ENST00000440465	ENST00000543053	NAMPTL
ENST00000448205	ENST00000493432	PLSCR1
ENST00000459891	ENST00000366658	ARV1
ENST00000535788	ENST00000302182	UBB
ENST00000288599	ENST00000395856	NCOA1
ENST00000277165	ENST00000340893	FAM120A
ENST00000336509	ENST00000355954	MFSD11
ENST00000375799	ENST00000375793	PLEKHM2
ENST00000396595	ENST00000381753	LIMCH1
ENST00000320560	ENST00000441606	ANO6
ENST00000508591	ENST00000510985	HELQ
ENST00000483871	ENST00000244020	SRSF6
ENST00000421373	ENST00000295760	FBLN2
ENST00000315032	ENST00000413835	MARK2
ENST00000399365	ENST00000336934	STARD13
ENST00000540001	ENST00000360494	TMEM48
ENST00000263433	ENST00000376393	PPP1R12C
ENST00000444203	ENST00000390687	SNRPN
ENST00000221431	ENST00000392076	SARS2
ENST00000354266	ENST00000394692	RHOT1
ENST00000320084	ENST00000449098	HNRNPC
ENST00000252015	ENST00000451813	TRPC4AP
ENST00000456430	ENST00000295989	CAND2
ENST00000311559	ENST00000540145	CYLD
ENST00000260130	ENST00000422546	SDCBP
ENST00000519664	ENST00000307662	SYNPO
ENST00000339159	ENST00000369496	ATP1A1
ENST00000343135	ENST00000357668	ASAP1
ENST00000344680	ENST00000430715	RTN2
ENST00000395860	ENST00000358490	METTL21D
ENST00000369095	ENST00000369094	C1orf51
ENST00000465950	ENST00000369367	SCAF11
ENST00000350766	ENST00000373498	MAPKAP1
ENST00000397865	ENST00000397860	MBP
ENST00000405010	ENST00000406072	HDAC9
ENST00000523357	ENST00000517975	ELP3
ENST00000541943	ENST00000395146	AFF1
ENST00000225928	ENST00000225929	HSD17B1
ENST00000377191	ENST00000539513	XRN2
ENST00000396445	ENST00000376451	KIAA1217
ENST00000403295	ENST00000402135	FANCL
ENST00000409411	ENST00000409957	PREPL
ENST00000355659	ENST00000455123	PON2
ENST00000549017	ENST00000548580	MYL6
ENST00000330439	ENST00000568293	MT1E
ENST00000507754	ENST00000252576	NDUFA13
ENST00000402521	ENST00000262414	EFTUD2
ENST00000313875	ENST00000381753	LIMCH1
ENST00000432329	ENST00000353267	CREB1
ENST00000375723	ENST00000370623	ARHGEF7
ENST00000380529	ENST00000380500	SERPINB6
ENST00000444696	ENST00000409411	PREPL
ENST00000537426	ENST00000544081	SEC62
ENST00000357710	ENST00000261970	PSEN1
ENST00000349223	ENST00000346183	NFATC3
ENST00000497011	ENST00000264381	BCHE
ENST00000539829	ENST00000370336	DDX43

SFIG 12

(a)

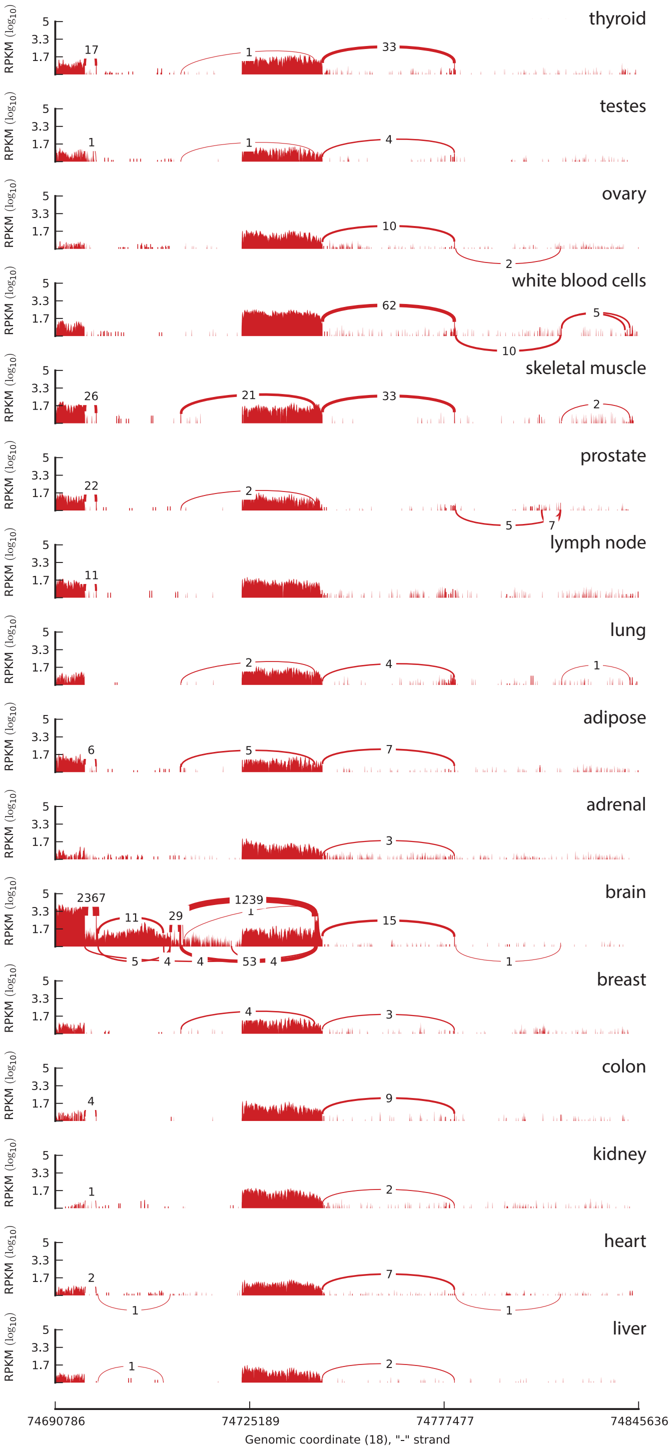
thyroid
testes
ovary
white blood cells
skeletal muscle
prostate
lymph node
lung
adipose
adrenal
brain
breast
colon
kidney
heart
liver
RPKM (log_{10})
5
3.3
1.7
74690786
74725189
74777477
74845636
Genomic coordinate (18), "-" strand

(b)

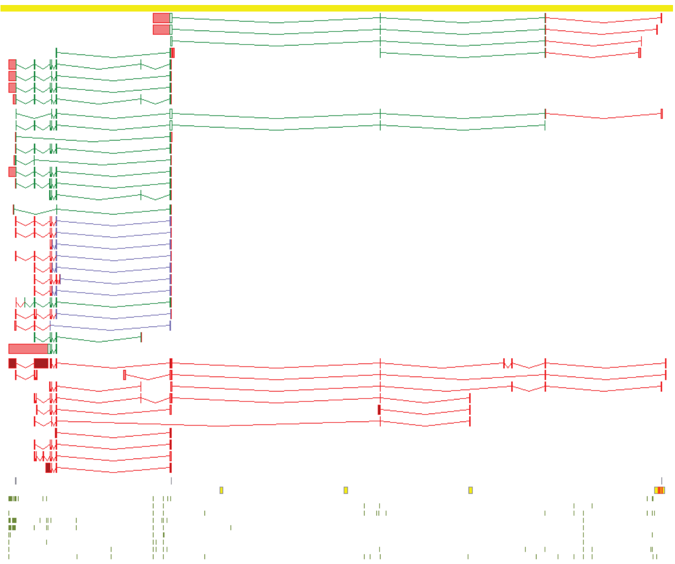

SFIG 13

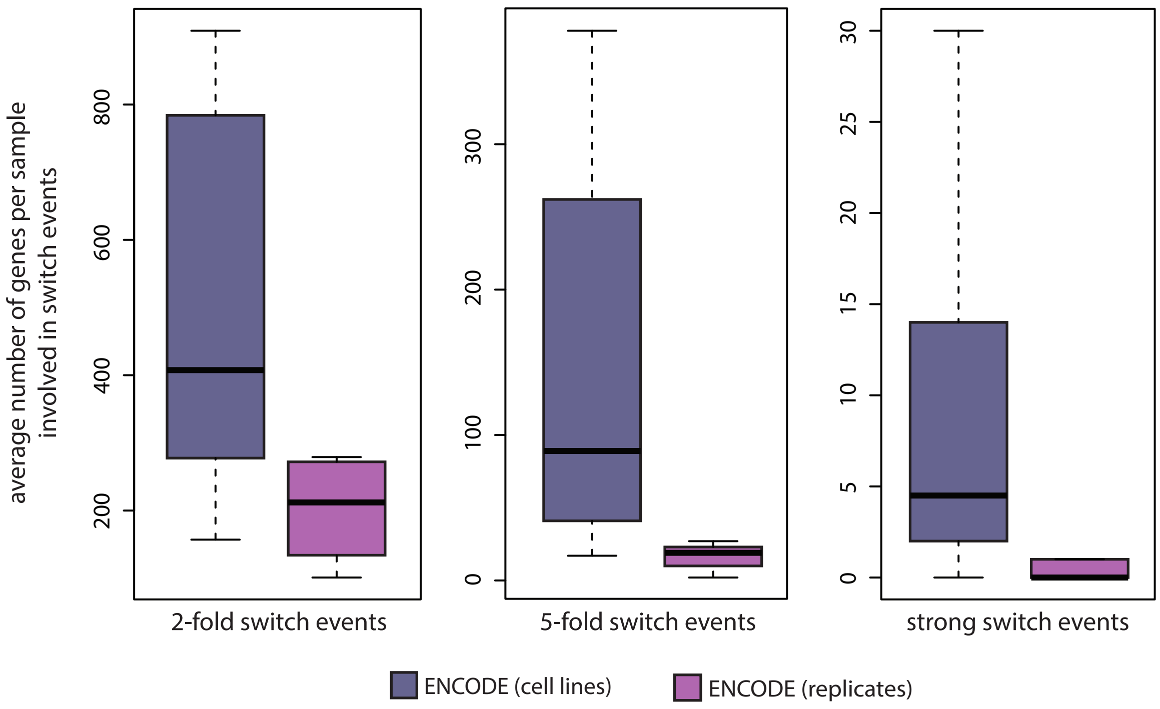
average number of genes per sample involved in switch events
200
400
600
800
2-fold switch events
0
100
200
300
5-fold switch events
0
5
10
15
20
25
30
strong switch events
ENCODE (cell lines)
ENCODE (replicates)

SFIG 14

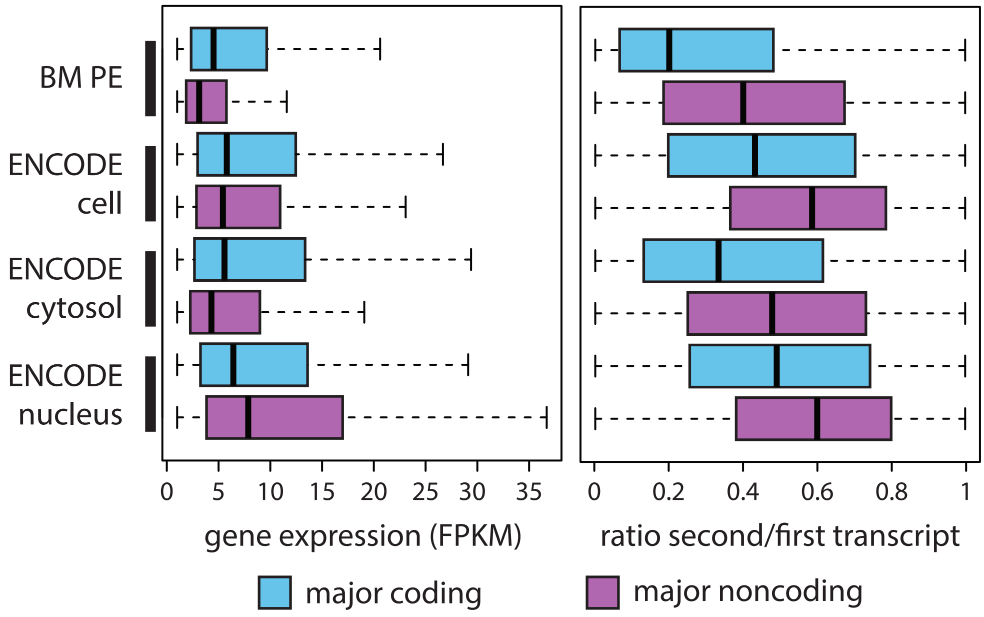
BM PE
ENCODE cell
ENCODE cytosol
ENCODE nucleus
0 5 10 15 20 25 30 35
gene expression (FPKM)
0 0.2 0.4 0.6 0.8 1
ratio second/first transcript
major coding
major noncoding

SFIG 15

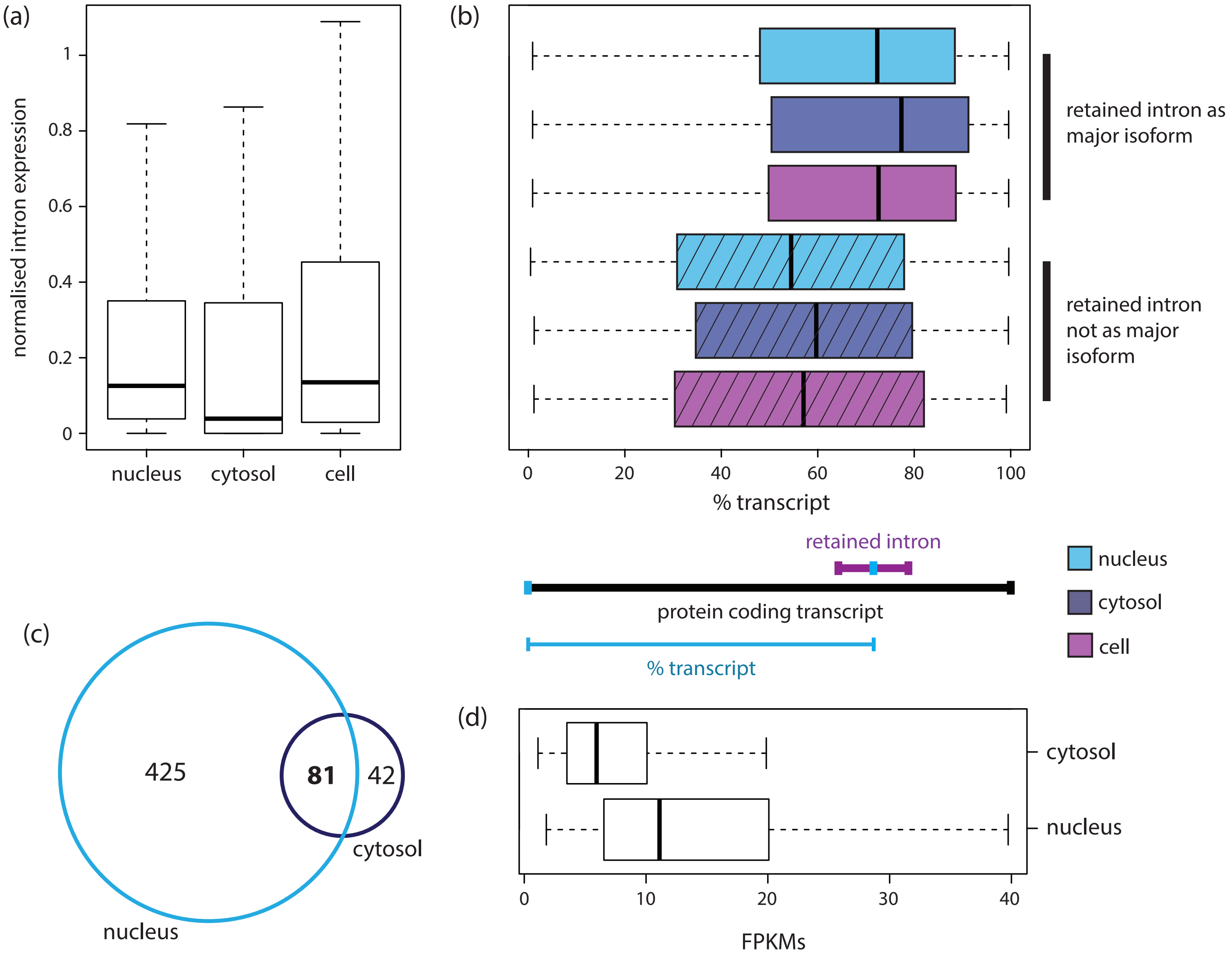

(a)
normalised intron expression
1
0.8
0.6
0.4
0.2
0
nucleus
cytosol
cell
(b)
retained intron as major isoform
retained intron not as major isoform
0
20
40
60
80
100
% transcript
retained intron
protein coding transcript
% transcript
nucleus
cytosol
cell
(c)
425
81
42
cytosol
nucleus
(d)
cytosol
nucleus
0
10
20
30
40
FPKMs

SFIG 16

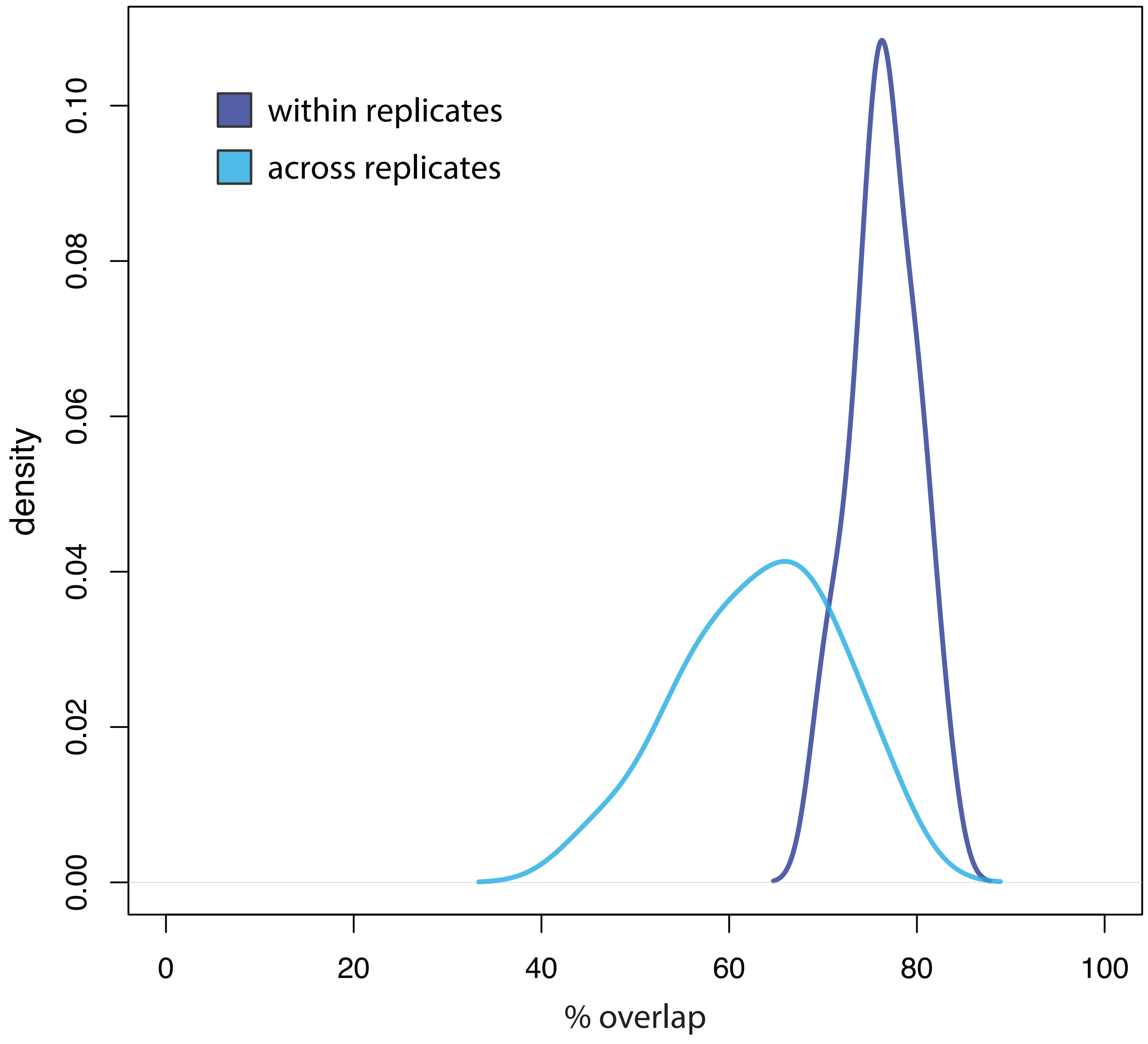

within replicates
across replicates
density
0.00
0.02
0.04
0.06
0.08
0.10
0
20
40
60
80
100
% overlap
