# Supplementary material for: Transcriptome analysis of human tissues and cell lines reveals one dominant transcript per gene
Source: Genome Biol. 2013 Jul 1;14(7):R70. doi: 10.1186/gb-2013-14-7-r70 (PMC4053754; doi:10.1186/gb-2013-14-7-r70)
Supplement: Additional file 3 — Supplementary Files [file gb-2013-14-7-r70-S3.ZIP › supp_files/supp_file_legends.pdf]

Supplementary Files

File S1: Genes for which the major transcript is not the longest one annotated (a) or does not contain the longest CDS (b). The lists include cases that were consistently detected both in primary tissues and cell lines.

File S2: Genes with a recurrent major transcript across tissues.

File S3: Genes with a recurrent major transcript across cell lines.

File S4: Switch events in tissues: 2-fold (a); 5-fold (b) and strong switch events (c).

File S5: Switch events in cell lines: 2-fold (a); 5-fold (b) and strong switch events (c).
