# Supplementary material for: Transcriptome analysis of human tissues and cell lines reveals one dominant transcript per gene
Source: Genome Biol. 2013 Jul 1;14(7):R70. doi: 10.1186/gb-2013-14-7-r70 (PMC4053754; doi:10.1186/gb-2013-14-7-r70)
Supplement: Additional file 4 — Supplementary Methods and Results [file gb-2013-14-7-r70-S4.PDF]

Supplementary Methods

Defining Biotype and Status

The biotype and status of a transcript are defined by the putative functional potential, structural properties (eg retained introns), similarity to entries in major sequence databases and type or length of the supporting evidence by which they are supported. The section below describes in more detail the criteria by which a transcript is classified. Locus level biotype is inferred from the individual biotypes of the transcripts it contains e.g. a protein-coding locus must contain at least one protein-coding transcript.

Biotype Definition: Protein-coding loci

- Protein coding: contains a CDS
- Nonsense-mediated decay (NMD): contains a CDS but has one or more splice junctions >50bp downstream of stop codon
- Non-stop decay (NSD): contains a CDS but has no stop codon before the polyA site is reached
- Processed transcript: does not contain a CDS that fulfills annotation criteria not necessarily functionally non-coding
- Retained intron: has retained intronic sequence compared to a reference variant with no strong evidence evidence (CAGE data, alternative ATG or polyA features, strong cross-species stop codon conservation) for function

Supplementary Results

Overlap with APPRIS

In order to address the impact of our observations at the protein level, we used a set of transcripts predicted to be translated into proteins by an entirely independent method, based on protein domains, structures and conservation across species [43]. We quantified, in primary tissues, the overlap between APPRIS predictions and those transcripts that are always identified as major, expressed above 1 FPKM and coding. As a contrast, we also quantified the overlap for those transcripts that are never identified as major, expressed below 1 FPKM and noncoding. Focusing on common genes in these two scenarios (n=6,082), we detect an overlap of 45.61% for the first case vs 29.35% for the second. Further taking into consideration only those genes that are expressed in all tissues (n=1,682), the overlap is 59.93% for the first set and 22.06% for the second.

Major transcript expression patterns in cell lines

Similarly to the analyses performed with the primary tissue data, we sought to quantify the proportion of genes that express the same major transcript across different samples. From replicate comparison we observe an average overlap in the set of major transcripts of 76% (69.77-80.47%; SD=4.97; **¡Error! No se encuentra el origen de la referencia.**). This percentage increases up to 87% (83.49-91.94%; SD=3.25) in the cytosolic fraction and with an expression threshold of 10 FPKMs. As expected, the overlap detected within replicates is higher than across replicates (Additional file 1, Figure S16). Further focusing on genes that are expressed in at least two cell line samples (n=13,437), we identify 30% of those for which the major isoform is recurrently expressed (Additional file 2, Table S11 and Additional file 3, File S3). This percentage is higher for the cytosolic fraction and, similarly to primary tissues, increases when considering ubiquitously expressed genes and more stringent expression thresholds – e.g. we detect an overlap of 72% for ubiquitous genes that are expressed above 10 FPKM in the cytosol.
